# Dissociated *Pmch* and Cre Expression in Lactating *Pmch*-Cre BAC Transgenic Mice

**DOI:** 10.3389/fnana.2020.00060

**Published:** 2020-08-21

**Authors:** Bethany G. Beekly, William C. Frankel, Tova Berg, Susan J. Allen, David Garcia-Galiano, Giancarlo Vanini, Carol F. Elias

**Affiliations:** ^1^Department of Molecular and Integrative Physiology, University of Michigan, Ann Arbor, MI, United States; ^2^Neuroscience Graduate Program, University of Michigan, Ann Arbor, MI, United States; ^3^Baylor College of Medicine, Houston, TX, United States; ^4^Department of Anesthesiology, School of Medicine, University of Michigan, Ann Arbor, MI, United States; ^5^Department of Obstetrics and Gynecology, School of Medicine, University of Michigan, Ann Arbor, MI, United States

**Keywords:** preoptic area, VGAT, VGLUT2, hypothalamus, sex-differences

## Abstract

The melanin-concentrating hormone (MCH) system plays a role in many physiological processes including reproduction and lactation. However, research regarding the function of MCH on different aspects of the reproductive function lags, due in part to a lack of validated genetic models with which to interrogate the system. This is particularly true in the case of female reproduction, as the anatomy and function of the MCH system is not well-characterized in the female mouse. We set out to determine whether the commercially available *Pmch*-Cre transgenic mouse line is a viable model to study the role of MCH neurons in distinct female reproductive states. We found that *Pmch* is transiently expressed in several nuclei of the rostral forebrain at the end of lactation. This includes the medial subdivision of the medial preoptic nucleus, the paraventricular nucleus of the hypothalamus, the ventral subdivision of the lateral septum, the anterodorsal preoptic nucleus and the anterodorsal nucleus of the thalamus. The *Pmch* expression in these sites, however, does not reliably induce Cre expression in the *Pmch*-Cre (BAC) transgenic mouse, making this line an inadequate model with which to study the role of MCH in behavioral and/or neuroendocrine adaptations of lactation. We also contribute to the general knowledge of the anatomy of the murine MCH system by showing that lactation-induced *Pmch* expression in the rostral forebrain is mostly observed in GABAergic (VGAT) neurons, in contrast to the typical MCH neurons of the tuberal and posterior hypothalamus which are glutamatergic (VGLUT2).

## Introduction

The melanin-concentrating hormone (MCH) system has been implicated in a diverse array of fundamental physiological processes such as metabolic regulation, stress response, and sleep (Baker et al., 1985; Bahjaoui-Bouhaddi et al., 1994; Qu et al., 1996; Verret et al., 2003). Several studies have provided compelling evidence that MCH neurons also regulate aspects of reproductive function such as the release of luteinizing hormone (LH) and lactation (Knollema et al., 1992; Attademo et al., 2006).

While MCH neurons have widespread projections throughout the brain, they originate from a discrete region of the hypothalamus (Bittencourt et al., 1992; Elias and Bittencourt, 1997; Bittencourt and Elias, 1998; Elias et al., 1998; Casatti et al., 2002; Sita et al., 2007). In rats, MCH expression is almost exclusively restricted to cell bodies in the incertohypothalamic area (IHy, aka rostromedial zona incerta or ZIm), the lateral hypothalamic area (LHA), and the perifornical area (PFx). The distribution of MCH neurons in mice and rats – and, indeed, in all rodents in which the system has been studied – follows a similar basic plan. However, close study of the mouse and rat reveals some key differences, including a population of periventricular MCH neurons seen in rats but not in mice, and the absence of more posterior groups in the mouse (Bittencourt et al., 1992; Diniz et al., 2019). Interestingly, MCH is transiently observed in the medial preoptic area (MPO), the periventricular preoptic nucleus, and the most rostral parts of the paraventricular nucleus of the hypothalamus (PVH) of female rats during lactation (Knollema et al., 1992; Rondini et al., 2010) and, to a lesser extent, in the MPO of the mouse (Knollema et al., 1992; Rondini et al., 2010; Diniz et al., 2019). The role of MPO MCH expression in the lactating rodents is unclear, potentially due to a lack of validated models with which to study this phenomenon. Indeed, functional studies in mice lag behind the anatomy of the MCH system, as the majority of the experiments referenced up to this point which involve reproductive function were performed in rats.

In the present study, we sought to determine whether the commercially available Tg(*Pmch*-cre)1Lowl/J (“*Pmch*-Cre”) transgenic mouse line (Kong et al., 2010) is a viable model to interrogate the role of the MCH system in female physiology. The *Pmch*-Cre mouse was developed using a bacterial artificial chromosome (BAC) containing the coding region for Cre recombinase flanked by upstream and downstream regulatory elements of the *Pmch* gene (Kong et al., 2010). This technique is moderately efficient and used to be a common method of introducing DNA sequences in the genome of a model organism. However, the loci of insertion for the BAC is random: it is incorporated into a genomic site with a unique set of regulatory elements, potentially imposing additional layers of regulation on Cre expression. It may also fail to recapitulate epigenetic regulation that occurs at the native gene locus and relies on enhancers that may be located hundreds of thousands of bases up- or downstream (Dean, 2006; Krivega and Dean, 2012). The unpredictable nature of this method necessitates thorough validation, which has not been performed in female mice, particularly in lactating dams.

In addition to defining the locations of MCH expression during lactation, we sought to determine whether these MCH neurons potentially release γ-aminobutyric acid (GABA), glutamate, both, or neither of the classical fast neurotransmitters. We used brain tissue from Cre-driven reporter lines expressing GFP in neurons expressing the vesicular glutamate transporter (VGLUT2) or the vesicular GABA transporter (VGAT) to verify the fast neurotransmitter phenotype of MCH neurons in different brain regions.

## Materials and Methods

### Mice

#### Animal Care

Mice were housed in a vivarium at the University of Michigan with a 12/12 light/dark cycle and *ad libitum* access to food and water. The mice received phytoestrogen-reduced Envigo diet 2016 (16% protein/4% fat), except during breeding when mice were fed phytoestrogen-reduced Envigo diet 2019 (19% protein/8% fat). Phytoestrogen-reduced diet is routinely used in our laboratory to avoid the effects of exogenous estrogens on mouse physiology. All procedures and experiments were carried out in accordance with the guidelines established by the National Institutes of Health Guide for the Care and Use of Laboratory Animals and approved by the University of Michigan Committee on Use and Care of Animals (Animal Protocol #8712).

#### *Pmch*-Cre;eGFP Mice

Commercially available *Pmch*-Cre [Tg(*Pmch*-Cre)1Lowl/J, JAX^®^; stock #014099] mice were used. This mouse model was developed using a BAC containing a Cre coding sequence downstream of the mouse pro-melanin concentrating hormone (*Pmch*) promoter (Kong et al., 2010).

The *Pmch*-Cre mice were crossed with two different mouse lines carrying Cre-inducible reporter genes. First, *Pmch*-Cre mice were crossed with the B6;129-*Gt(ROSA)26Sor^*tm*2*Sho*^*/J line (JAX^®^; stock #004077, “R26-eGFP”). This mouse carries a targeted mutation of the *R26* locus with a loxP-flanked transcriptional blocking cassette preventing the expression of CAG promoter-driven enhanced green fluorescent protein (eGFP) reporter (Mao et al., 2001). Cre-mediated excision of the blocking cassette in *Pmch*-Cre;R26-eGFP mice results in expression of GFP in cells expressing Cre.

*Pmch*-Cre mice were also crossed with the B6;129S4-*Gt(ROSA)26Sor^*tm*9(EGFP/Rpl10*a)Amc*^*/J line (JAX^®^; stock #024750, “eGFP-L10a”), kindly donated by Dr. David Olson (University of Michigan), to generate *Pmch*-Cre;eGFP-L10a reporter mice. The eGFP-L10a mice express a chimeric L10a ribosomal subunit fused to eGFP. Successful Cre-mediated excision results in expression of the eGFP:L10A fusion protein in Cre-expressing tissues (Liu et al., 2014). Due to the ribosomal eGFP fusion, this mouse model enables a strong fluorescent signal to accumulate in cell bodies.

#### Vgat-Cre;eGFP and Vglut2-Cre;eGFP Mice

Vgat-IRES-Cre:*Slc32a1^*tm*2(cre)Lowl/J^* (JAX^®^ mice, Stock #016962) and Vglut2-IRES-Cre: S*lc17a6^*tm*2(cre)Lowl/^*J (JAX^®^ mice, Stock #016963) mice were crossed with eGFP-L10a reporter to generate mice which express GFP in VGAT- or VGLUT2-expressing cells, respectively (Vgat-Cre;eGFP-L10a and Vglut2-Cre;eGFP-L10a mice) (Vong et al., 2011).

#### Genotyping

All mice were genotyped before experiments by extracting DNA from tail tip biopsies using the REDExtract-N-Amp^TM^ Tissue PCR Kit, catalog no. XNAT (Sigma-Aldrich^®^). PCR was performed with a Bio-Rad C1000^TM^ Thermal Cycler and included positive (DNA from the original JAX mice) and negative (sterile water) controls. Primer sequences can be found in Table 1.

**TABLE 1:** Primer sequences used for genotyping mice and performing radioactive *in situ* hybridization.

Primer	Sequence
**Pmch-Cre (genotyping)**	
Common	GAA AAG ATA AGG CCT TCA AGT GCT
Internal Positive Control Reverse	GAT CTT TCT GCA GTA TCT TCC TTC
Transgene Reverse	ATC GAC CGG TAA TGC AGG CAA
**R26-eGFP (genotyping)**	
Forward	AAGTTCATCTGCACCACCG
Reverse	TCCTTGAAGAAGATGGTGCG
**L10-eGFP (genotyping)**	
Forward 1	GAG GGG AGT GTT GCA ATA ACC
Forward 2	TCT ACA AAT GTG GTA GAT CCA GGC
Reverse	CAG ATG ACT ACC TAT CCT CCC
**Pmch cDNA Template (ISH)**	
Forward	(CAG AGA TGC AAT TAA CCC TCA CTA AAG GGA GA) AGC ATC AAA CTA AGG ATG GCA
Reverse	(CCA AGC CCT CTA ATA CGA CTC ACT ATA GGG AGA) GCA TAC ACC TGA GCA TGT CAA AA

### Experimental Groups

Sexually naïve adult male *Pmch*-Cre;eGFP mice (8–12 weeks of age) were used. Adult female mice were divided into sexually naïve (8–10 weeks of age) and lactating groups (16–18 weeks of age) for a total of three experimental groups (*n* = 4 per group). An additional group of females (*n* = 4) was evaluated 5 days after weaning of the offspring as control for the lactation group. Experimental groups were evaluated in both reporter lines: *Pmch*-Cre;R26-eGFP and *Pmch*-Cre;eGFP-L10a.

Additionally, Vgat-Cre;eGFP-L10a and Vglut2-Cre;eGFP-L10a dams were bred with Vglut2-Cre and Vgat-Cre males to generate two more lactation groups in which to assess the fast neurotransmitter phenotype of MCH neurons (*n* = 3 per group).

### Perfusion and Histology

Adult male *Pmch*-Cre;eGFP mice were euthanized and brains harvested between 8 and 12 weeks of age. Sexually naïve females, 8–10 weeks of age, were ascertained to be in diestrus before tissue was harvested. Brains were collected from lactating females on day 19 of lactation (16–18 weeks of age).

All mice were anesthetized with isoflurane and transcardially perfused with saline prepared with diethyl pyrocarbonate (DEPC)-treated water followed by 10% neutral buffered formalin for 10 min. Following perfusion, brains were dissected and postfixed in 20% sucrose/10% buffered formalin overnight at 4°C, then cryoprotected in 20% sucrose in DEPC-treated 1× phosphate buffered saline (PBS) overnight at 4°C. Brains were sectioned with a freezing Leica SM2010 R microtome (4 series, 30-μm thickness, in the frontal plane). Sections were stored at −20°C in RNAse-free cryoprotectant (20% glycerol, 30% ethylene glycol in DEPC-PBS).

### Radioactive *in situ* Hybridization

Single-label radioactive *in situ* hybridization (ISH) was performed on one series of brain sections from each animal. Sections were mounted onto RNAse-free SuperFrost Plus slides (Fisher Scientific). Mounted tissue was fixed in 4% paraformaldehyde in DEPC-treated 1× PBS for 20 min, dehydrated in increasing concentrations of ethanol, cleared of lipids using xylenes, and subsequently rehydrated in decreasing concentrations of ethanol. The slides were pretreated by microwaving in sodium citrate buffer (pH 6.0), then dehydrated again in increasing concentrations of ethanol, air-dried, and stored at −20°C (Sibony et al., 1995; Frazão et al., 2013; Mohsen et al., 2017).

The *Pmch* cDNA (template) was produced from mouse hypothalamic RNA and used to amplify a 478 bp sequence in the coding region of the *Pmch* gene (IDT, Inc.). Primer sequences are delineated in Table 1. The *Pmch* riboprobe was generated by *in vitro* transcription using ^35^S-UTP as the radioisotope. ^35^S-labeled *Pmch* probe was diluted in hybridization solution (50% formamide, 10 mM Tris-HCl pH 8.0, 0.01% of yeast tRNA, 0.05% of total yeast RNA, 10 mM dithiothreitol/DTT, 10% dextran sulfate, 0.3 M NaCl, 1 mM EDTA, and 1× Denhardt’s solution) and applied to slides, which were allowed to hybridize overnight at 55°C, as routinely done by our group (Frazão et al., 2013). Following post-hybridization treatment with RNAse, stringency washes with saline sodium citrate (SSC) buffer and dehydration in increasing concentrations of ethanol, sections were dried at room temperature and slides were placed in X-ray film cassettes with BMR-2 film (Kodak, Rochester, NY, United States) for 1 day (tuberal and posterior levels of the hypothalamus) or 4 days (rostral forebrain). Slides were then dipped in NTB autoradiographic emulsion (Kodak), dried for 3 h and stored in light-protected slide boxes at 4°C for 6 days (tuberal and posterior levels of the hypothalamus) or 14 days (rostral forebrain). Slides were developed with Dektel developer (Kodak, VWR, Radnor, PA, United States), dehydrated in increasing concentrations of ethanol, cleared in xylenes and coverslipped with DPX mounting medium (Electron Microscopy Sciences).

### Dual-Label Immunohistochemistry

Dual-label immunohistochemistry (IHC) for MCH or NEI and GFP was performed in one series of brain sections from each animal. Sections were blocked for 30 min in 3% normal donkey serum (NDS) and incubated overnight in chicken anti-GFP (1:10,000, Aves Labs, AB_2307317) and either rabbit anti-MCH (1:5,000, Phoenix Pharmaceuticals, AB_2722682) or rabbit anti-NEI (1:15,000, kindly provided by Dr. Paul Sawchenko, Salk Institute for Biological Studies, La Jolla, CA, United States) followed by secondary antisera (goat anti-chicken conjugated to Alexa Fluor^TM^ 488 AB_2534096, and donkey anti-rabbit conjugated to Alexa Fluor^TM^ 594 AB_141637) for 1 h (1:500, Thermo Fisher Scientific). Sections were mounted onto gelatin-coated slides, air-dried, and coverslipped with Fluoromount G mounting medium (Electron Microscopy Sciences).

In one series of brain sections from lactating dams (*n* = 3), a tyramide signal amplification (TSA) procedure was used. Briefly, sections were incubated for 10 min in 10% H_2_O_2_ to block endogenous peroxidase activity, followed by a 1 h blocking step in 3% NDS. They were incubated overnight in rabbit anti-MCH (1:30,000). The next day, sections were incubated for 1 h in donkey anti-rabbit biotinylated secondary antibody (1:500, Jackson ImmunoResearch Laboratories AB_2340593) followed by 1 h in avidin-biotin complex (ABC) solution (1:500, Vector Laboratories). Finally, they were incubated in tyramide reagent (1:250, Perkin Elmer) + 0.003% H_2_O_2_ followed by 30 min in AlexaFluor^TM^ 594-conjugated streptavidin (1:1,000, Thermo Fisher Scientific) before mounting and coverslipping with FluoroMount G mounting medium.

### Dual Label *in situ* Hybridization (ISH) and Immunohistochemistry (IHC)

Dual-label ISH and IHC were performed to determine the colocalization of Pmch mRNA and Vgat-Cre or Vglut2-Cre GFP-ir (Garcia-Galiano et al., 2017). Briefly, free-floating sections from lactating females (*n* = 3/genotype) were treated with 0.1% sodium borohydride for 15 min and 10 min with 0.25% acetic anhydride in DEPC-treated 0.1 M triethanolamine (TEA, pH 8.0). Sections were incubated overnight at 50°C in the hybridization solution containing the ^35^S-*Pmch* riboprobe. Subsequently, sections were treated with RNase A for 30 min and submitted to stringency washes in sodium chloride-sodium citrate buffer (SSC). Sections were blocked (3% BSA in PBS-Triton) then incubated with anti-GFP antibody (1:5,000) overnight at 4°C. Sections were incubated for 1.5 h in a goat anti-chicken AlexaFluor 488 antibody and mounted onto SuperFrost plus slides. After overnight air drying, slides were dehydrated in increasing concentrations of ethanol and dipped in NTB-2 autoradiographic emulsion (Kodak/Carestream) to reflect the company merge/transition, dried and stored in light-protected boxes at 4°C for 3 weeks. Finally, slides were developed with D-19 developer (Kodak), dehydrated in graded ethanol, cleared in xylene, and coverslipped with DPX mounting medium.

### Fluorescent *in situ* Hybridization

Fluorescent ISH for *Pmch* and *Slc32a1* was performed using the RNAscope^®^ fluorescent multiplex detection Kit (Advanced Cell Diagnostics, cat. no. 320850) as previously described (Wang et al., 2012). Briefly, mice were deeply anesthetized with isoflurane and decapitated. Brains were collected and immediately frozen on dry ice, then cut into six series of 16-μm sections on a cryostat at −20°C and mounted onto Superfrost Plus slides. Slides were heated at 60°C, then immersed in 10% buffered formalin for 2 h at 4°C. They were dehydrated in increasing concentrations of ethanol, cleared in xylenes, and rehydrated in decreasing concentrations of ethanol. Next, slides were boiled for 10 min in sodium citrate buffer and incubated for 10 min in 0.03% sodium dodecyl sulfate. Slides were subsequently dried at room temperature and a hydrophobic barrier was drawn around the sections. From this point forward, steps were carried out in humidity control trays to prevent sections from drying. Endogenous peroxidase activity was blocked with a 10-min incubation with H_2_O_2_ followed by rinsing with nuclease-free water. RNAscope protease III was then applied and slides were heated for 30 min at 40°C before once again rinsing with nuclease-free water. Hybridization was performed by applying pre-warmed target positive and negative control probes to sections and heating for 2 h at 40°C. Then, the amplification of each probe (AMP 1-2) was performed sequentially, incubating for 30 min at 40°C then rinsing in wash buffer after each Multiplex FL v2 Amp solution. The slides were developed using two RNAscope Multiplex FL v2 HRP-C*n* solutions where *n* = 1–2 (one probe per channel). For each channel, this was performed by incubating in RNAscope Multiplex FL v2 HRP-C*n* for 15 min at 40°C, rinsing with wash buffer, incubating for 30 min at 40°C with TSA + Fluorescein (Akoya Biosciences, cat. no. SKU NEL741001KT), incubating in RNAscope Multiplex FL v2 HRP blocker for 15 min at 40°C, then rinsing once more with wash buffer. Finally, slides were counterstained with DAPI and coverslipped with ProLong Gold antifade mounting medium (ThermoFisher Scientific).

### Quantitative Analysis of Colocalization

Slides from each cohort of *Pmch*-Cre/GFP mice were examined under an Axio Imager M2 Microscope or a SteREO DiscoveryV8 (Zeiss). The digital Allen Mouse Brain Atlas was used as a reference to determine relative location within the hypothalamus and identify the primary sites containing MCH-immunoreactivity, GFP-immunoreactivity, and *Pmch* mRNA expression. Images were acquired with a digital camera (Axiocam, Zeiss) using Zen software. All sections were examined and single- and dual-labeled cells were quantified in 20× magnification using ImageJ with the Cell Counter plugin in one representative section of each area of interest (i.e., IHy, PFx, and LHA). For data illustration, only sharpness, contrast, and brightness were adjusted.

## Results

### *Pmch* Expression and MCH Immunoreactivity in Tuberal and Posterior Hypothalamus Are Similar in Naïve Male, Female, and Lactating Mice

**FIGURE 1:**
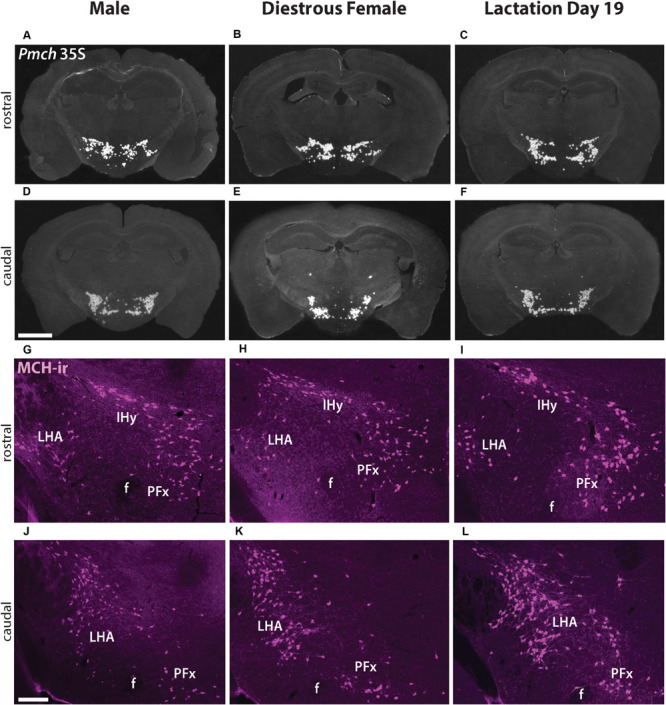
*Pmch* expression and MCH-immunoreactivity (-ir) (magenta, pseudocolored) in the tuberal hypothalamus are similar in naïve male, sexually naïve diestrous female, and lactating mice. **(A–F)** Dark field micrographs showing the distribution of *Pmch* mRNA (silver grains) in rostral **(A–C)** and caudal **(D–F)** aspects of the tuberal hypothalamus in all three groups of mice: sexually naïve male **(A,D)**, diestrous female **(B,E)**, and lactating **(C,F)** mice. **(G–L)** Fluorescent micrographs showing the distribution of MCH-ir in cells of the rostral **(G–I)** and caudal **(J–L)** aspects of the tuberal hypothalamus in sexually naïve male **(G,J)**, diestrous female **(H,K)** and lactating **(I,L)** mice. f, fornix; IHy, incertohypothalamic area; LHA, lateral hypothalamic area; PFx, perifornical area. Scale bar: **(A–F)** 1,000μm and **(G–L)** 200 μm.

*In situ* hybridization for *Pmch* was performed in brain sections from sexually naïve males and females in diestrus to visualize the distribution of *Pmch* mRNA and assess whether it is sexually dimorphic and/or distinct in lactating females. Intense hybridization signal was observed in males in cells of the IHy, LHA, and PFx with virtually no signal elsewhere in the brain. Populations of MCH cells were identified in the IHy as characterized by their relatively dorsal location within the hypothalamus, just below the thalamus. Populations of MCH cells were defined in the LHA and PFx using other nearby structures including the fornix, optic tract, and internal capsule. There was no difference in the distribution pattern of *Pmch* mRNA between the two groups that could be visually ascertained (Figures 1A,B,D,E). Furthermore, the distribution of *Pmch* mRNA in IHy, LHA, and PFx of the lactating dams was also similar to that of the sexually naïve male and female mouse (Figures 1C,F).

To assess if the distribution of the MCH peptide is similar in all three groups, MCH-immunoreactivity (MCH-ir) was examined and compared in sexually naïve male and female as well as lactating female mouse brains. Abundant MCH-ir was observed in perikarya and fibers in the IHy, LHA, and PFx, in all groups in accordance with the mRNA pattern and previous studies (Bittencourt et al., 1992; Diniz et al., 2019). No clear difference in the distribution pattern of MCH-ir was observed between groups (Figures 1G–L).

### A Subset of GFP+ Neurons in the Perifornical Area (PFx) Does Not Express MCH-ir

**FIGURE 2:**
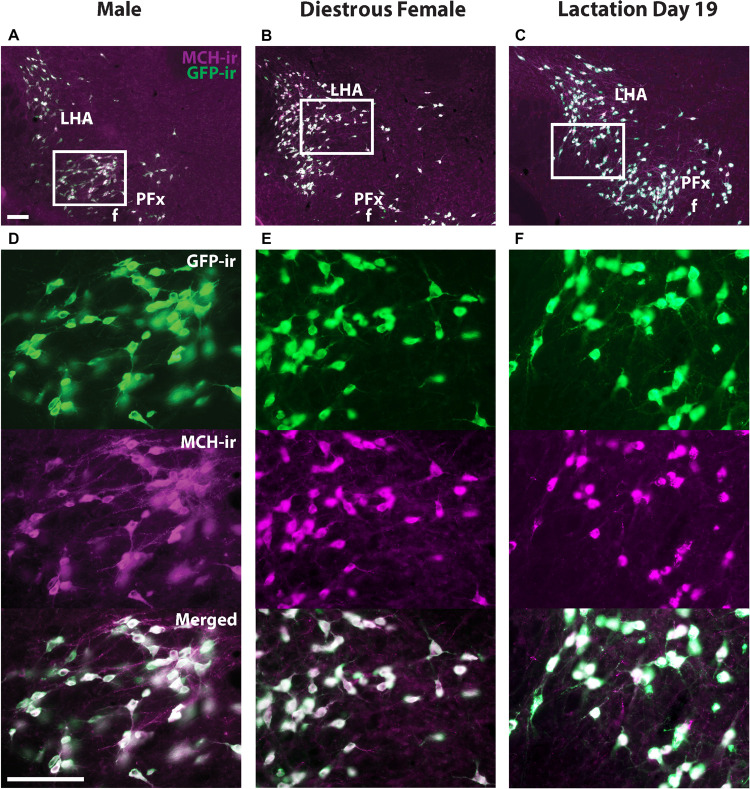
The majority of GFP+ neurons in the hypothalamus of *Pmch*-Cre;eGFP mice express MCH-immunoreactivity (-ir). Fluorescent micrographs showing the colocalization of GFP-ir and MCH-ir in the lateral hypothalamus of *Pmch*-Cre;eGFP-L10a male **(A)**, sexually naïve diestrus **(B)** and lactating (day 19) mice **(C)**. Scale bars: 100 μm.

**FIGURE 3:**
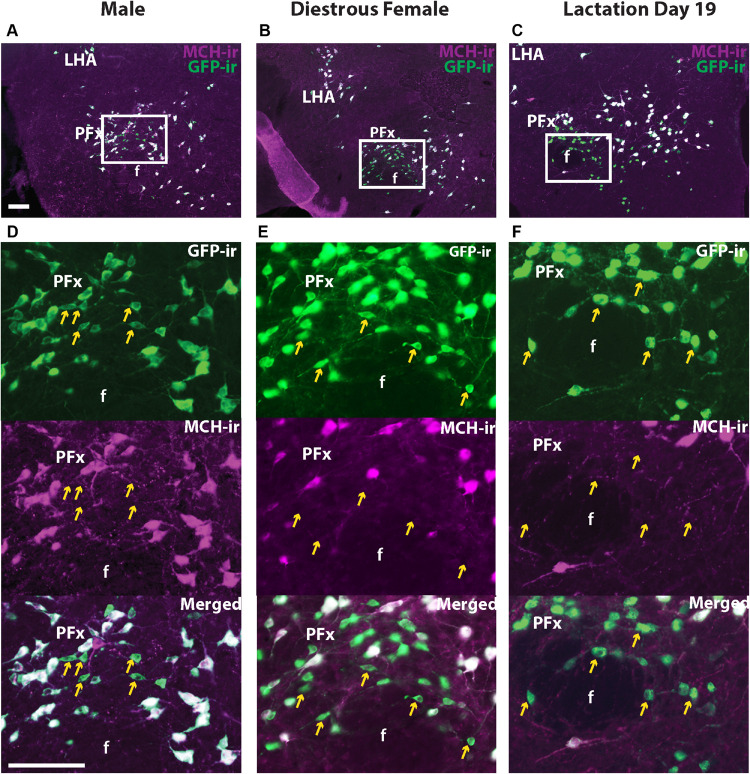
A subset of GFP+ neurons in the perifornical area (PFx) does not express MCH-ir. Fluorescent micrographs showing GFP-ir and MCH-ir in the perifornical area (PFx) of a *Pmch*-Cre;eGFP male **(A)**, diestrus female **(B)**, and lactating dam **(C)**. Note the presence of a subset of GFP-ir neurons in all groups that do not express MCH-ir (arrows). Sections are between 74 and 77 of 132 (Allen Mouse Brain Atlas, 2004). f, fornix LHA, lateral hypothalamic area; PFx, perifornical area. Scale bars: 100 μm.

Melanin-concentrating hormone- and Cre-induced GFP-ir were compared in sexually naïve male and female as well as lactating *Pmch*-Cre;R26-eGFP mouse brains. Virtually all GFP+ neurons expressed MCH-ir in the IHy and LHA (Figure 2). However, a population of GFP+ neurons that do not express MCH-ir was consistently observed in the PFx of all groups of mice. To assess whether this was an artifact of our reporter gene, we repeated this experiment with a different reporter line. In *Pmch*-Cre;eGFP-L10a mice, the same population of GFP+/MCH− cells was observed in all three groups (Figure 3). These neurons are primarily found lateral to the fornix at the level of the tuberal division of the LHA. The GFP+/MCH− cells have a distinctive small, circular morphology and cluster together near the fornix. A few such neurons were occasionally observed in other nuclei of the hypothalamus, primarily in the dorsal IHy, as well as more rostral regions such as the medial septum and prefrontal cortex, but this expression is inconsistent.

**FIGURE 4:**
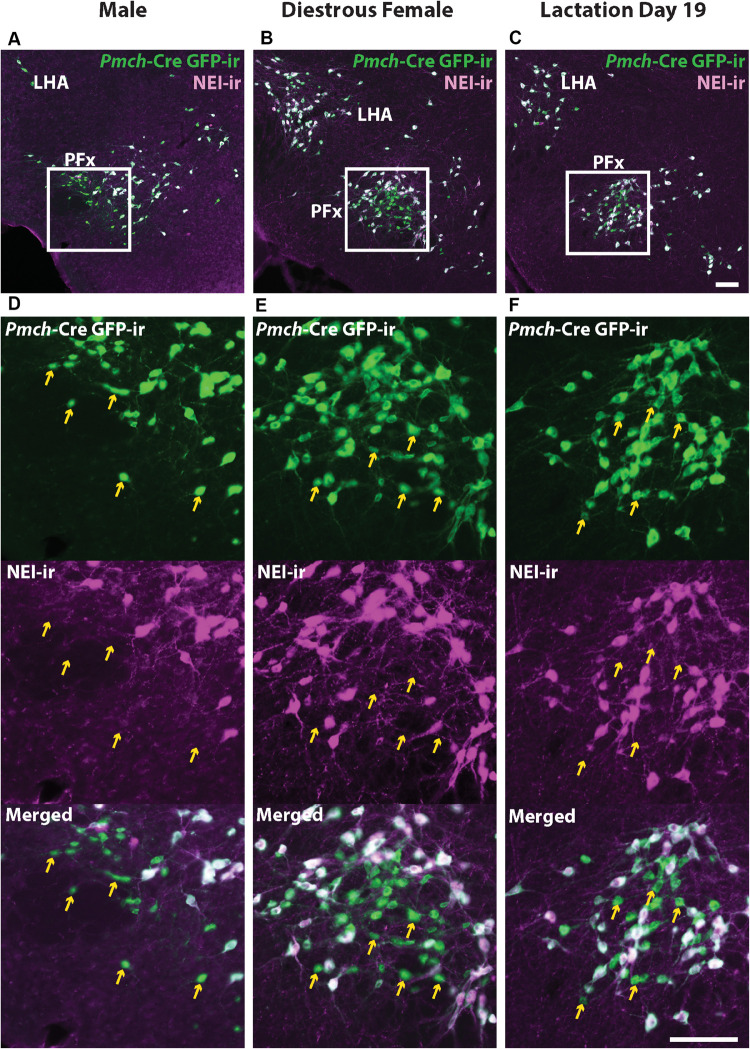
GFP+ /MCH- neurons in the perifornical area (PFx) do not express NEI-ir. Fluorescent micrographs showing GFP-ir and NEI-ir in the perifornical area (PFx) of a *Pmch*-Cre;eGFP male **(A)**, diestrus female **(B)**, and lactating dam **(C)**. Note the presence of a subset of GFP-ir neurons in all groups that do not express NEI-ir (arrows) which appears to correspond to the similar group of neurons observed that express GFP-ir but not MCH-ir. Sections are between 74 and 77 of 132 (Allen Mouse Brain Atlas, 2004). f, fornix LHA, lateral hypothalamic area; PFx, perifornical area. Scale bars **(A–D)** and **(E–F)**: 100 μm.

MCH is just one of several peptide products of the *Pmch* transcript, the other major one being NEI, so we evaluated NEI- and GFP-ir to determine whether the PFx GFP+/MCH− neuronal population expresses NEI. Abundant NEI-ir was observed in virtually all GFP-ir perikarya and fibers in the IHy, and LHA of all three groups, showing that, as reported in rats (Bittencourt et al., 1992), virtually all hypothalamic MCH neurons also contain NEI. However, a population of small GFP immunoreactive neurons in the PFx was also negative for NEI-ir (Figure 4). This GFP+/NEI− subset of neurons presumably overlaps with the population of GFP+/MCH− neurons previously described (Figure 3).

### Expression of *Pmch* and Cre-Induced GFP-ir Are Dissociated in Several Nuclei of the Rostral Forebrain in Lactating Dams

**FIGURE 5:**
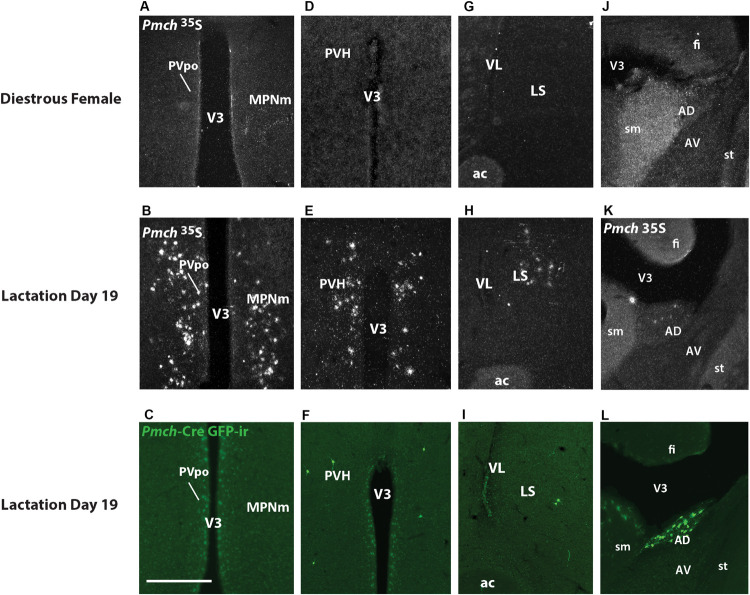
Expression of Pmch and Cre-induced GFP-ir are dissociated in several nuclei of the rostral forebrain in lactating dams. **(A)** Dark field photomicrograph of the medial preoptic nucleus (MPN) of a sexually naïve female in diestrus. **(B)** Dark field photomicrograph of the MPN of a lactation day 19 female. **(C)** Fluorescent photomicrograph of the MPN of a lactation day 19 female. **(D)** Dark field photomicrograph of the paraventricular nucleus (PVH) of a sexually naïve female in diestrus. **(E)** Dark field photomicrograph of the PVH of a lactation day 19 female. **(F)** Fluorescent photomicrograph of the PVH of a lactation day 19 female. **(G)** Dark field photomicrograph of the lateral septum (LS) of a sexually naïve female in diestrus. **(H)** Dark field photomicrograph of the LS of a lactation day 19 female. **(I)** Fluorescent photomicrograph of the LS of a lactation day 19 female. **(J)** Dark field photomicrograph of the anterodorsal thalamic nucleus (AD) of a sexually naïve female in diestrus. **(K)** Dark field photomicrograph of the AD of a lactation day 19 female. **(L)** Fluorescent photomicrograph of the AD of a lactation day 19 female. Images are taken from levels 54/132 (MPNm), 58/132 (PVH), 50/132 (LS), and 58/132 (AD) (Allen Mouse Brain Atlas, 2004). ac, anterior commissure; AV, anteroventral nucleus of the thalamus; fi, fimbria; sm, stria medullaris; st, stria terminalis; V3, third ventricle; VL, lateral ventricle. Scale bar **(A–L)**: 200 μm.

To further map Cre-induced GFP distribution, we initially performed a comprehensive analysis of *Pmch* expression in the rostral forebrain of sexually naïve females in diestrus and lactating (day 19) dams. As previously described in lactating rats, we observe *Pmch* expression in the MPO and PVH of lactating mice. However, the pattern of mRNA distribution was distinct comparing both murine species. At the level of the anterior commissure, *Pmch* expression is observed in the MPO of lactating but not sexually naïve mice and is restricted to the periventricular nucleus and medial subdivision of medial preoptic nucleus (MPNm) with some lateral spreading below and above to include the anterodorsal preoptic nucleus (ADP) and the principal subdivision of the bed nucleus of the stria terminalis (BSTpr) (Figures 5A,B). *Pmch* mRNA was also observed in the rostral PVH of lactating but not sexually naïve mice (Figures 5D,E) and inconsistently in forebrain regions where it has not been reported in the rat. We found a moderate to dense population of *Pmch* cells in the lateral septum (LS) of lactating but not sexually naïve mice (Figures 5G,H) and the anterodorsal nucleus of the thalamus (AD) just lateral to the stria medullaris (Figures 5J,K).

**FIGURE 6:**
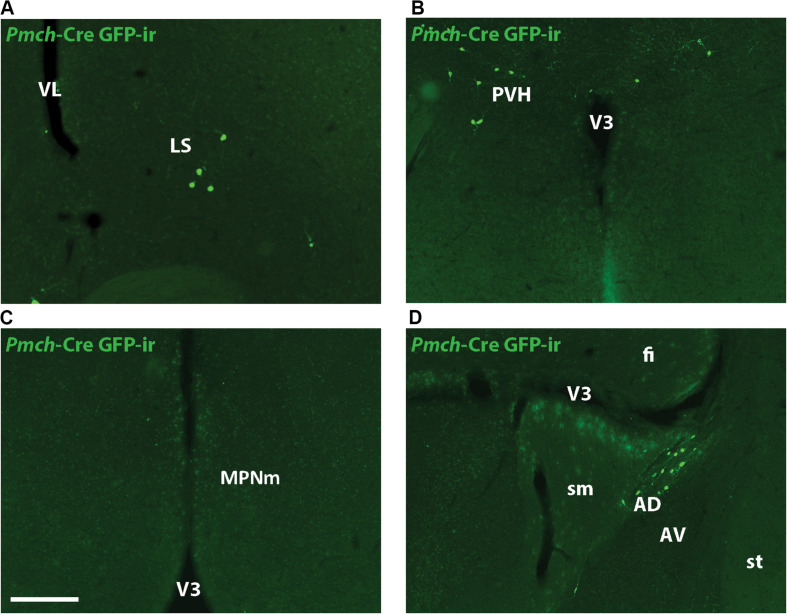
Cre-induced GFP expression in lactation-specific regions of the rostral forebrain is unchanged post-lactation. Fluorescent micrographs showing GFP-ir in the LS **(A)**, PVH **(B)**, MPNm **(C)**, and AD **(D)** of female mice five days post-weaning. Images are taken from levels 54/132 (MPNm), 50/132 (LS), and 58/132 (PVH/AD) (Allen Mouse Brain Atlas). AD, anterodorsal nucleus of the thalamus; AV, anteroventral nucleus of the thalamus; fi, fimbria; LS, lateral septum; MPNm, medial preoptic nucleus, medial part; sm, stria medullaris; st, stria terminalis; V3, third ventricle; VL, lateral ventricle. Scale bar **(A–D)**: 200 μm.

GFP-ir was examined in rostral forebrain of lactating *Pmch*-Cre;R26-eGFP and *Pmch*-Cre;eGFP-L10a dams to ascertain whether *Pmch* mRNA expression induces Cre expression in these regions. The latter reporter strain was preferred because the GFP fluorescence was more intense. Despite this more intense signal, little to no GFP-ir was observed in the MPO (Figure 5C) and inconsistent GFP-ir was observed in the PVH, with some animals showing strong expression while others had almost none (Figure 5F). This contrasts with the gene expression data, which shows high *Pmch* mRNA expression in these regions. Clear GFP-ir was detected in the LS (Figure 5I), and AD (Figure 5L). To assess if a delay in Cre and GFP expression was the cause of this inconsistency, a group of females was perfused 5 days after weaning (or 7 days post lactation day 19). No differences in eGFP-ir was observed compared to females perfused at lactation day 19 (Figure 6). The additional time for recombination and GFP expression did not yield higher GFP-ir. Collectively, our findings indicate that *Pmch* and Cre expression are incongruent: a large number of *Pmch* neurons do not express GFP-ir and a group of GFP+ neurons in the dorsal MS does not express *Pmch*.

### Expression of VGLUT2 and VGAT Differs in Subsets of *Pmch* Neurons

To gain insights into the fast neurotransmitter phenotype of MCH neurons we used Vgat- and Vglut2-Cre;eGFP-L10a reporter mice, dual label ISH and immunohistochemistry and dual label ISH.

**FIGURE 7:**
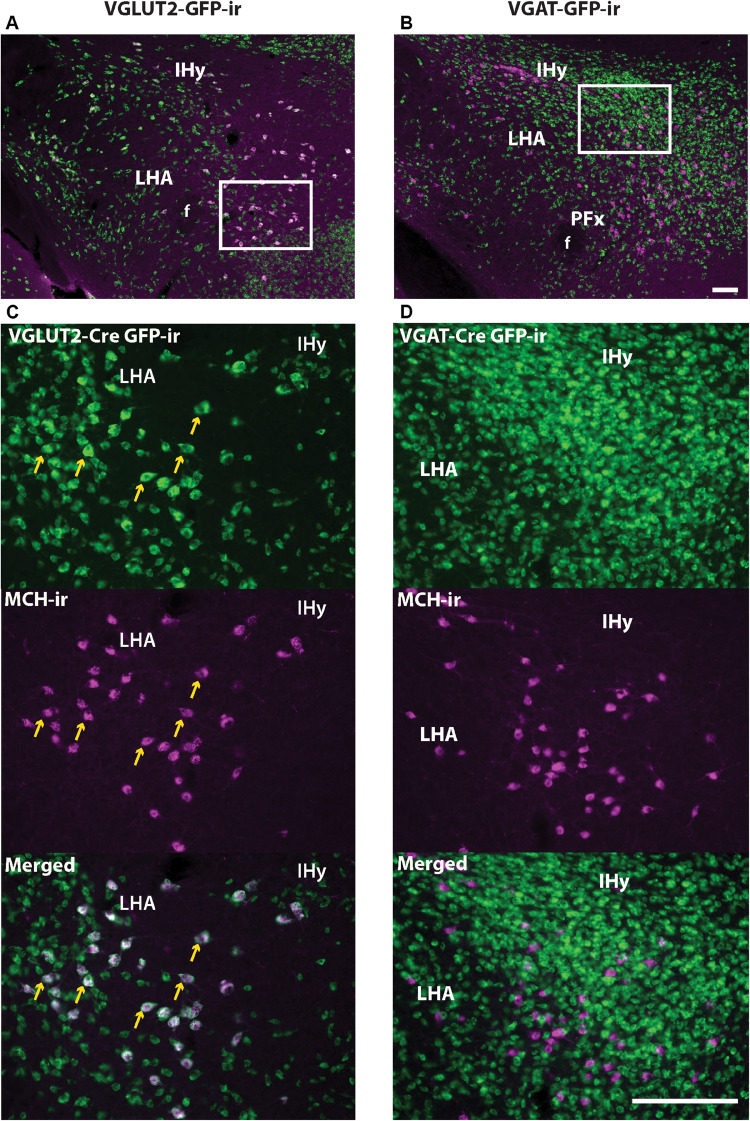
MCH- and Vglut2-Cre GFP-immunoreactivity (-ir) are co-expressed in neurons of the tuberal and posterior hypothalamus. **(A,C)** Immunohistochemistry for MCH and GFP in the lateral hypothalamus of Vglut2-Cre;eGFP mice shows that MCH-ir neurons predominantly colocalize with VGLUT2 (arrows). **(B,D)** Immunohistochemistry for MCH and GFP in the lateral hypothalamus of Vgat-Cre;eGFP mice shows that MCH-ir neurons do not colocalize with VGAT. Images depict tissue from postpartum females on lactation day 19, but the same result was observed in sexually naïve males and females. IHy, incertohypothalamic area; LHA, lateral hypothalamic area. Scale bars **(A,B)** and **(C,D)**: 100 μm.

Dual immunofluorescence was performed in brains from Vgat- and Vglut2-Cre;eGFP-L10a reporter mice to assess the colocalization of MCH-ir and GFP-ir in sexually naïve males and females in diestrus, and in nursing dams on day 19 of lactation. In the LHA, IHy, and PFx, as previously suggested by single-cell RNA sequencing data (Mickelsen et al., 2019), MCH-ir in all groups colocalized with Vglut2-Cre;eGFP (Figures 7A,C). Very few or virtually no MCH immunoreactive cells colocalized with Vgat-Cre;eGFP (Figures 7B,D).

**FIGURE 8:**
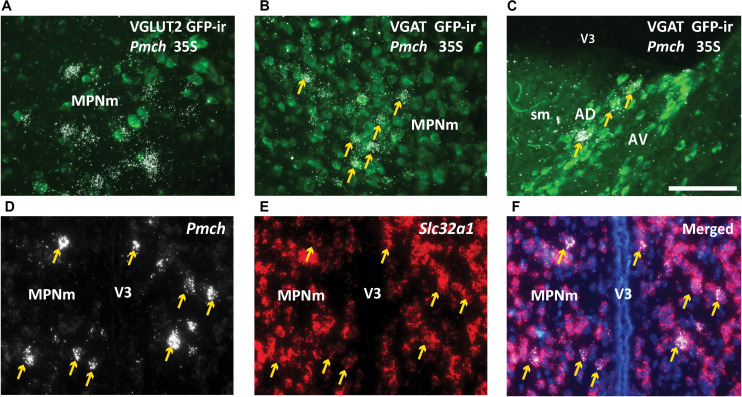
*Pmch* is expressed in rostral forebrain neurons that co-express *Slc32a1* (VGAT). **(A,B)** Fluorescent and dark field micrographs showing the majority of *Pmch* in the MPN do not colocalize with VGLUT2 **(A)** but do colocalize with VGAT **(B)**. **(C)** Fluorescent and dark field micrograph showing the majority of *Pmch* in the AD also colocalizes with VGAT. **(D–F)** Fluorescent micrographs showing co-expression (arrows) of *Pmch* (white) and *Slc32a1* (red) in medial aspect of the medial preoptic nucleus (MPNm) using RNAscope^®^ technology. DAPI counterstain in blue. AD, anterodorsal nucleus of the thalamus; AV, anteroventral nucleus of the thalamus; MPNm, medial preoptic nucleus, medial part; sm, stria medullaris; VL, lateral ventricle; V3, third ventricle. Scale bars **(A–F)**: 100 μm.

In the rostral forebrain of lactating dams, MCH-ir and NEI-ir were low and the expression observed was highly inconsistent between animals. The *Pmch*-Cre induced GFP-ir that was observed did not label *Pmch*-expressing neurons. To evaluate co-expression with the *Pmch* transcript, we used brain sections from lactating Vgat- and Vglut2-Cre;eGFP reporter mice. ISH for *Pmch* in combination with immunohistochemical amplification of GFP fluorescent signal was performed in all three mouse groups. In the LHA, IHy, and PFx, *Pmch* mRNA in all groups of mice colocalized with Vglut2-Cre;eGFP primarily, and sporadically with Vgat-Cre;eGFP, consistent with our immunohistochemical data. However, in the lactating dams, colocalization was less clear. *Pmch* mRNA in the MPO, PVH, LS, and AD colocalized primarily with Vgat-Cre;eGFP and not Vglut2-eGFP (Figures 8A–C). Because this result contradicts recent studies (Teixeira et al., 2019), we performed fluorescent ISH using RNAscope to further examine colocalization of *Pmch* with *Slc32a1* (VGAT). The findings were unambiguous and demonstrated that virtually all *Pmch* expressing neurons co-express *Slc32a1* (Figures 8D–F). In summary, our findings indicate that MCH neurons in the LHA, PFx, and IHy are overwhelmingly glutamatergic, and those in the rostral forebrain induced during lactation are mostly GABAergic.

## Discussion

In this study, we assessed if the commercially available Tg(*Pmch*-cre)1Lowl/J transgenic mouse line (Kong et al., 2010) is a viable model to interrogate the role of the MCH system in the female reproductive function. We found that in male and female (diestrous and lactating) mice, a group of Cre-induced GFP-expressing cells that do not express MCH- or NEI-ir is consistently observed in the PFx. In lactating dams, *Pmch* expression is observed in the MPO and anterior PVH, and in previously unreported forebrain nuclei, i.e., LS and AD. However, most of these sites do not show Cre-induced GFP-ir in the Tg(*Pmch*-cre)1Lowl/J transgenic mouse line. We also found that typical MCH neurons in the tuberal and posterior hypothalamus co-express VGLUT2 (*Slc17a6*) whereas lactation-induced *Pmch* expression in rostral forebrain is mostly observed in VGAT (*Slc32a1*) neurons.

This Tg(*Pmch*-cre)1Lowl/J mouse model shares many similarities with the C57BL/6-Tg(*Pmch*-cre)1Rck/J line. Both lines utilize a BAC transgene. The promoter for the C57BL/6-Tg(*Pmch*-cre)1Rck/J line is somewhat larger, including 108 kb upstream of the MCH gene in the BAC regulatory element as opposed to the 64 kb in the Tg(*Pmch*-cre)1Lowl/J. The strains also have nearly identical penetrance and specificity (Kong et al., 2010; Jego et al., 2013). Ultimately, we chose to focus on the Tg(*Pmch*-cre)1Lowl/J line because it is used and cited more frequently, making our findings applicable to more researchers in the field (Kong et al., 2010; Varin et al., 2018; Dilsiz et al., 2020).

Whereas most previous studies have been focused on peptide distribution, here we gave special attention to the distribution and expression of the *Pmch* gene. As previously described for MCH-ir, *Pmch* expression is very similar in naïve male and female mice with clear anterior and posterior patterns of distribution (Diniz et al., 2019). The anterior pattern of mRNA expression is characterized by distinct populations of neurons in the IHy, PFx, and a few neurons in the LHA (anterior division), while the posterior pattern primarily consists of dense expression in the LHA (tuberal division) and PFx. We have further shown that virtually all MCH-ir cells express GFP (and thus Cre recombinase) in the *Pmch*-Cre reporter mouse, indicating that Cre expression in the Tg(*Pmch*-Cre)1Lowl/J BAC transgenic mouse line is highly penetrant. It is worth mentioning that we observe higher penetrance than what was demonstrated in the originating publication (Kong et al., 2010). This is likely due to the use of a ribosomal protein-associated reporter line, which is more concentrated in the soma, whereas the original publication validated the model used a TdTomato reporter, which is expressed in fibers and thus yields a more diluted signal (Madisen et al., 2010; Liu et al., 2014).

However, we observe some GFP-ir cells which do not express MCH located in the PFx. The cause for this discrepancy is not evident, but an artifact (ectopic expression) of the BAC transgene is a reasonable explanation, calling into question the specificity of Cre expression. Alternatively, MCH could play a developmental role in these cells. If its production is turned off by adulthood, MCH-ir and *Pmch* would not be detected by our assays, yet GFP signal would still be visible as it is driven by a ubiquitous promoter. Future work will be necessary to address this question by characterizing hypothalamic *Pmch* mRNA and MCH peptide expression at various timepoints in the developing mouse brain.

In lactating mice, *Pmch* expression is clearly different from rats (Knollema et al., 1992). While in rats, *Pmch* expression is triggered at mid-lactation and becomes dense in the MPN and PVH, in mice, *Pmch* is observed only at the end of lactation (day 19) at low to moderate levels specifically in the MPNm (Teixeira et al., 2019) and anterior PVH. Notably, the mice also show lactation-induced *Pmch* in previously unreported brain sites, including the LS, the ADP, the ventral BSTpr and the anterior AD. The role of MCH system in these sites is not known. Furthermore, the inconsistency of Cre-mediated GFP expression indicate that the *Pmch*-Cre BAC transgenic mouse model is not useful for investigating transient MCH expression in lactating dams. Euthanizing lactating dams 5 days after weaning the offspring to allow more time for Cre-mediated recombination and GFP expression did not change the low to non-existent levels of GFP-ir in the MPNm, PVH and LS of the lactating dam. Researchers should also note that dams euthanized on lactating day 19 or 7 days post-weaning exhibit ectopic GFP expression in brain sites we did not observe *Pmch* mRNA. Use of female breeders in studies of MCH/NEI function may generate unreliable data.

Turning on MCH production for just several days at the end of lactation suggests that its role is highly specific and time-sensitive. If this is the case, it may indicate that MCH in these neurons is subjected to unique epigenetic regulation that may not be reproduced in the BAC sequence. Moreover, differences at the level of transcription, such as the use of enhancers, which inform MCH production and processing could interfere with Cre expression in these cells if the enhancers are not located within the BAC transgene. A BAC genomic clone containing 64 kb upstream and 34 kb downstream of *Pmch* gene was used to generate the original MCH-Cre mice with the goal of capturing most of the gene’s regulatory elements (Kong et al., 2010). However, mammalian enhancers can be located as far as one million base pairs from the transcriptional start site of the gene in question (Dean, 2006; Maston et al., 2006; Krivega and Dean, 2012). Thus, it is possible that important enhancer activity has been excluded from the BAC regulatory element.

It has been suggested that prolactin-induced phosphorylation of STAT5 (pSTAT5) has a role in the lactation-induced MCH-ir in the MPO. STAT5 is a known activator protein for hundreds of enhancers, particularly those implicated in pregnancy- and lactation-associated genes (Yamaji et al., 2013; Teixeira et al., 2019). The seven STAT family proteins are all believed to target the same palindromic core motif, TTC*N_2__–__4_*GAA. STAT5A and B in particular have a strong preference for palindromic core motifs of *N*_3_ (Ehret et al., 2001). A brief search using DNASTAR Lasergene software shows that approximately 50 sites per 100 kb contain sequence TTC*N*_3_GAA, yielding close to 500 potential pSTAT5 binding sites upstream of the *Pmch* gene regulatory element that are potentially missing from the BAC regulatory elements. Whereas these are clearly speculations, it is possible that regulatory sequences essential for *Pmch* expression during lactation are not included in the BAC used in this mouse model.

For the purposes of anatomical and specific functional studies, i.e., viral injections, tract tracing or colocalization with other transcripts and peptides, this may be an acceptable model because nearly all of the Cre-expressing cells are indeed MCH-positive neurons with the exception of the small, highly localized population of PFx neurons that are easy to identify.

It has long been debated whether MCH neurons release GABA, glutamate, both, or neither of the classical fast neurotransmitters. In rats, it has been documented that MCH neurons of the tuberal hypothalamus express *Gad1* (glutamic acid decarboxylase or GAD67), the enzyme that catalyzes the decarboxylation of glutamate to GABA, and the vesicular GABA transporter (VGAT) can be found in MCH terminals (Sapin et al., 2010; del Cid-Pellitero and Jones, 2012). However, glutamate has been observed in MCH cells and demonstrated to be released from MCH terminals (Chee et al., 2015; Schneeberger et al., 2018). Using a different reporter system, however, others claim that these neurons are neither GABAergic nor glutamatergic (Blanco-Centurion et al., 2019). The glutamatergic-leaning transcriptional profile of hypothalamic MCH neurons has largely been reinforced with the availability of advanced genomic techniques, i.e., single-cell RNA sequencing (Mickelsen et al., 2019). Our data using distinct methodologies is in agreement with the latter and demonstrates that the typical MCH neurons in the tuberal hypothalamus are essentially glutamatergic. It is also worth mentioning that our findings are not contradictory to previous studies in rats showing that MCH neurons co-express GAD67. Species differences should not be ignored as the colocalization of MCH and GAD67 or lack thereof in mice has not been shown. The presence of GAD67 indicates that the cell has the necessary machinery to synthetize GABA from glutamate (Roberts and Frankel, 1950; Martin, 1987). Whether this process is observed in specific physiological state(s) in MCH neurons of the mouse hypothalamus needs further investigation.

Transient MCH expression in the rat MPO, on the other hand, has been shown to occur in GAD67 expressing neurons (Rondini et al., 2010). Using immunohistochemistry and radioactive ISH we show that transient lactation-induced MCH expression is largely restricted to VGAT + neurons, though in the MPO sporadic *Pmch* mRNA can apparently be also found in VGLUT2 + neurons. RNAscope confirms that most of lactation-induced *Pmch* neurons co-express *Slc32a1* (VGAT). This contrast in fast neurotransmitter phenotype could underlie fundamental differences in the function and properties of these transient MCH neurons as compared with their counterparts elsewhere in the hypothalamus. However, it also contradicts the recent finding that MCH neurons in the LHA, PFx, and IHy express neither Vglut2, Vglut3, nor Vgat (Teixeira et al., 2019). As aforementioned, this difference may arise from our use of an eGFP-L10a reporter strain rather than a TdTomato reporter. Because L10 is a ribosomal protein, it concentrates in the cytoplasm for ease of visualization whereas TdTomato also labels terminals, diluting the signal (Madisen et al., 2010; Liu et al., 2014).

We conclude that the *Pmch*-Cre mouse model is unsuitable for studying the role of MCH during lactation. Any Cre-dependent manipulations will not target the neurons which transiently express MCH during lactation, especially in the MPNm, PVH and LS, where the mRNA/GFP-ir discrepancy is most pronounced. This presents a need to develop other methods for investigating these neurons, which could involve the development of another transgenic mouse line. It would be particularly beneficial to use a knock-in strategy to increase specificity and congruence of Cre and *Pmch* expression in both sexes and all physiological states.

### Resource Identification Initiative

RRID described in methods session.

### Life Science Identifiers

This study uses genetically modified mouse models purchased from JAX mice. The nomenclature is in agreement with the International Committee on Standardized Genetic Nomenclature for Mice.

## Data Availability Statement

Requests to access the datasets should be directed to cfelias@umich.edu.

## Ethics Statement

The animal study was reviewed and approved by IACUC, University of Michigan.

## Author Contributions

BB, WF, and CE designed the study. BB and WF carried out the experiments. TB designed and generated the **Pmch** probe for radioactive **in situ** hybridization. DG-G assisted with probe design and execution of radioactive **in situ** hybridization. SA contributed with maintenance and genotyping of the *Pmch*-Cre mouse colony. GV contributed with maintenance and genotyping of the Vgat- and Vglut2-Cre;eGFP mouse colonies. BB analyzed data and compiled the manuscript. CE, GV, and SA contributed to manuscript revisions. All authors contributed to the article and approved the submitted version.

## Conflict of Interest

The authors declare that the research was conducted in the absence of any commercial or financial relationships that could be construed as a potential conflict of interest.

## References

[B1] Allen Mouse Brain Atlas (2004). *Allen Institute for Brain Science. Allen Mouse Brain Atlas, version 2 2011.* Available online at: https://mouse.brain-map.org/ (accessed July 1, 2020).

[B2] Attademo A. M. Rondini T. A. Rodrigues B. C. Bittencourt J. C. Celis M. E. Elias C. F. (2006). Neuropeptide glutamic acid-isoleucine may induce luteinizing hormone secretion via multiple pathways. *Neuroendocrinology.* 83 313–324. 10.1159/000096052 17016031

[B3] Bahjaoui-Bouhaddi M. Fellmann D. Griffond B. Bugnon C. (1994). Insulin treatment stimulates the rat melanin-concentrating hormone-producing neurons. *Neuropeptides* 27 251–258. 10.1016/0143-4179(94)90006-x 7808598

[B4] Baker B. I. Bird D. J. Buckingham J. C. (1985). Salmonid melanin-concentrating hormone inhibits corticotrophin release. *J. Endocrinol.* 106 R5–R8. 2991410 10.1677/joe.0.106r005

[B5] Bittencourt J. C. Elias C. F. (1998). Melanin-concentrating hormone and neuropeptide EI projections from the lateral hypothalamic area and zona incerta to the medial septal nucleus and spinal cord: a study using multiple neuronal tracers. *Brain Res.* 805 1–19. 10.1016/s0006-8993(98)00598-8 9733903

[B6] Bittencourt J. Presse F. Arias C. Peto C. Vaughan J. Nahon J. L. (1992). The melanin−concentrating hormone system of the rat brain: an immuno−and hybridization histochemical characterization. *J. Comp. Neurol.* 319 218–245. 10.1002/cne.903190204 1522246

[B7] Blanco-Centurion C. Luo S. Spergel D. J. Vidal-Ortiz A. Oprisen S. Van den Pol A. N. (2019). Dynamic network activation of hypothalamic mch neurons in rem sleep and exploratory behavior. *J. Neurosci.* 39 0305–0319. 10.1523/JNEUROSCI.0305-19.2019 PMC6670248 31036764

[B8] Casatti C. A. Elias C. F. Sita L. V. Frigo L. Furlani V. C. Bauer J. A. (2002). Distribution of melanin-concentrating hormone neurons projecting to the medial mammillary nucleus. *Neuroscience* 115 899–915. 10.1016/s0306-4522(02)00508-0 12435428

[B9] Chee M. J. Arrigoni E. Maratos-Flier E. (2015). Melanin-concentrating hormone neurons release glutamate for feedforward inhibition of the lateral septum. *J. Neurosci.* 35 3644–3651. 10.1523/jneurosci.4187-14.2015 25716862 PMC6605558

[B10] Dean A. (2006). On a chromosome far, far away: LCRs and gene expression. *Trends Genet.* 22 38–45. 10.1016/j.tig.2005.11.001 16309780

[B11] del Cid-Pellitero E. Jones B. E. (2012). Immunohistochemical evidence for synaptic release of GABA from melanin-concentrating hormone containing varicosities in the locus coeruleus. *Neuroscience* 223 269–276. 10.1016/j.neuroscience.2012.07.072 22890079

[B12] Dilsiz P. Aklan I. Sayar Atasoy N. Yavuz Y. Filiz G. Koksalar F. (2020). MCH neuron activity is sufficient for reward and reinforces feeding. *Neuroendocrinology* 110 258–270. 10.1159/000501234 31154452

[B13] Diniz G. Battagello D. Cherubini P. Reyes-Mendoza J. Luna-Illades C. Klein M. (2019). Melanin-concentrating hormone peptidergic system: comparative morphology between muroid species. *J. Comp. Neurol.* 527 2973–3001. 10.1002/cne.24723 31152440

[B14] Ehret G. B. Reichenbach P. Schindler U. Horvath C. M. Fritz S. Nabholz M. (2001). DNA binding specificity of different STAT proteins. Comparison of in vitro specificity with natural target sites. *J. Biol. Chem.* 276 6675–6688. 10.1074/jbc.m001748200 11053426

[B15] Elias C. F. Bittencourt J. C. (1997). Study of the origins of melanin-concentrating hormone and neuropeptide EI immunoreactive projections to the periaqueductal gray matter. *Brain Res.* 755 255–271. 10.1016/s0006-8993(97)00104-2 9175893

[B16] Elias C. F. Saper C. B. Maratos-Flier E. Tritos N. A. Lee C. Kelly J. (1998). Chemically defined projections linking the mediobasal hypothalamus and the lateral hypothalamic area. *J. Comp. Neurol.* 402 442–459. 10.1002/(sici)1096-9861(19981228)402:4<442::aid-cne2>3.0.co;2-r 9862320

[B17] Frazão R. Cravo R. M. Donato J. Ratra D. V. Clegg D. J. Elmquist J. K. (2013). Shift in Kiss1 cell activity requires estrogen receptor α. *J. Neurosci.* 33 2807–2820. 10.1523/jneurosci.1610-12.2013 23407940 PMC3713640

[B18] Garcia-Galiano D. Borges B. C. Donato J. Allen S. J. Bellefontaine N. Wang M. (2017). PI3Kα inactivation in leptin receptor cells increases leptin sensitivity but disrupts growth and reproduction. *JCI Insight* 12:e96728. 10.1172/jci.insight.96728 PMC5752267 29212950

[B19] Jego S.D. Glasgow S. Gutierrez Herrera C. Ekstrand M. Reed S. Boyce R. (2013). Optogenetic identification of a rapid eye movement sleep modulatory circuit in the hypothalamus. *Nat Neurosci.* 16 1637–1643. 10.1038/nn.3522 24056699 PMC4974078

[B20] Knollema S. Brown E. R. Vale W. Sawchenko P. E. (1992). Novel hypothalamic and preoptic sites of prepro-melanin-concentrating hormone messenger ribonucleic Acid and Peptide expression in lactating rats. *J. Neuroendocrinol.* 4 709–717. 10.1111/j.1365-2826.1992.tb00222.x 21554658

[B21] Kong D. Vong L. Parton L. E. Ye C. Tong Q. Hu X. (2010). Glucose stimulation of hypothalamic MCH neurons involves K(ATP) channels, is modulated by UCP2, and regulates peripheral glucose homeostasis. *Cell Metab.* 12 545–552. 10.1016/j.cmet.2010.09.013 21035764 PMC2998191

[B22] Krivega I. Dean A. (2012). Enhancer and promoter interactions-long distance calls. *Curr. Opin. Genet. Dev.* 22 79–85. 10.1016/j.gde.2011.11.001 22169023 PMC3342482

[B23] Liu J. Krautzberger A. M. Sui S. H. Hofmann O. M. Chen Y. Baetscher M. (2014). Cell-specific translational profiling in acute kidney injury. *J. Clin. Invest.* 124 1242–1254. 10.1172/jci72126 24569379 PMC3938273

[B24] Madisen L. Zwingman T. A. Sunkin S. M. Oh S. W. Zariwala H. A. Gu H. (2010). A robust and high-throughput Cre reporting and characterization system for the whole mouse brain. *Nat. Neurosci.* 13 133–140. 10.1038/nn.2467 20023653 PMC2840225

[B25] Mao X. Fujiwara Y. Chapdelaine A. Yang H. Orkin S. H. (2001). Activation of EGFP expression by Cre-mediated excision in a new ROSA26 reporter mouse strain. *Blood* 97 324–326. 10.1182/blood.v97.1.324 11133778

[B26] Martin D. L. (1987). Regulatory properties of brain glutamate decarboxylase. *Cell Mol. Neurobiol.* 7 237–253. 10.1007/bf00711302 3326683 PMC11567405

[B27] Maston G. A. Evans S. K. Green M. R. (2006). Transcriptional regulatory elements in the human genome. *Annu. Rev. Genomics Hum. Genet.* 7 29–59. 10.1146/annurev.genom.7.080505.115623 16719718

[B28] Mickelsen L. E. Bolisetty M. Chimileski B. R. Fujita A. Beltrami E. J. Costanzo J. T. (2019). Single-cell transcriptomic analysis of the lateral hypothalamic area reveals molecularly distinct populations of inhibitory and excitatory neurons. *Nat. Neurosci.* 04 642–656. Epub 2019/03/11. 10.1038/s41593-019-0349-8 30858605 PMC7043322

[B29] Mohsen Z. Sim H. Garcia-Galiano D. Han X. Bellefontaine N. Saunders T. L. (2017). Sexually dimorphic distribution of Prokr2 neurons revealed by the Prokr2-Cre mouse model. *Brain Struct. Funct.* 222 4111–4129. 10.1007/s00429-017-1456-5 28616754 PMC5937125

[B30] Qu D. Ludwig D. S. Gammeltoft S. Piper M. Pelleymounter M. A. Cullen M. J. (1996). A role for melanin-concentrating hormone in the central regulation of feeding behaviour. *Nature* 380 243–247. 10.1038/380243a0 8637571

[B31] Roberts E. Frankel S. (1950). gamma-Aminobutyric acid in brain: its formation from glutamic acid. *J. Biol. Chem.* 187 55–63. 14794689

[B32] Rondini T. A. Donato J. Rodrigues B. C. Bittencourt J. C. Elias C. F. (2010). Chemical identity and connections of medial preoptic area neurons expressing melanin-concentrating hormone during lactation. *J. Chem. Neuroanat.* 39 51–62. 10.1016/j.jchemneu.2009.10.005 19913090

[B33] Sapin E. Bérod A. Léger L. Herman P. A. Luppi P. H. Peyron C. (2010). A very large number of GABAergic neurons are activated in the tuberal hypothalamus during paradoxical (REM) sleep hypersomnia. *PLoS One* 5:e11766. 10.1371/journal.pone.0011766 20668680 PMC2909908

[B34] Schneeberger M. Tan K. Nectow A. R. Parolari L. Caglar C. Azevedo E. (2018). Functional analysis reveals differential effects of glutamate and MCH neuropeptide in MCH neurons. *Mol. Metab.* 07 83–89. Epub 2018/05/08. 10.1016/j.molmet.2018.05.001 29843980 PMC6026325

[B35] Sibony M. Commo F. Callard P. Gasc J. (1995). Enhancement of mRNA in situ hybridization signal by microwave heating. *Lab. Invest.* 73 586–591. 7474931

[B36] Sita L. Elias C. Bittencourt J. (2007). Connectivity pattern suggests that incerto-hypothalamic area belongs to the medial hypothalamic system. *Neuroscience* 148 949–969. 10.1016/j.neuroscience.2007.07.010 17707116

[B37] Teixeira P. D. S. Wasinski F. Lima L. B. Frazão R. Bittencourt J. C. Donato J. (2019). Regulation and neurochemical identity of melanin-concentrating hormone neurons in the preoptic area of lactating mice. *J. Neuroendocrinol.* 32:e12818. 10.1111/jne.12818 31782183

[B38] Varin C. Luppi P. H. Fort P. (2018). Melanin-concentrating hormone-expressing neurons adjust slow-wave sleep dynamics to catalyze paradoxical (REM) sleep. *Sleep* 41 1–12. 10.1093/sleep/zsy068 29618134

[B39] Verret L. Goutagny R. Fort P. Cagnon L. Salvert D. Leger L. (2003). A role of melanin-concentrating hormone producing neurons in the central regulation of paradoxical sleep. *BMC Neurosci.* 9:19. 10.1186/1471-2202-4-19 12964948 PMC201018

[B40] Vong L. Ye C. Yang Z. Choi B. Chua S. Lowell B. B. (2011). Leptin action on GABAergic neurons prevents obesity and reduces inhibitory tone to POMC neurons. *Neuron.* 71 142–154. 10.1016/j.neuron.2011.05.028 21745644 PMC3134797

[B41] Wang F. Flanagan J. Su N. Wang L. C. Bui S. Nielson A. (2012). RNAscope: a novel in situ RNA analysis platform for formalin-fixed, paraffin-embedded tissues. *J. Mol. Diagn.* 14 22–29. 22166544 10.1016/j.jmoldx.2011.08.002 PMC3338343

[B42] Yamaji D. Kang K. Robinson G. W. Hennighausen L. (2013). Sequential activation of genetic programs in mouse mammary epithelium during pregnancy depends on STAT5A/B concentration. *Nucleic Acids Res.* 41 1622–1636. 10.1093/nar/gks1310 23275557 PMC3561979

